# Pharmacokinetics of islatravir in participants with moderate hepatic impairment

**DOI:** 10.1128/aac.01553-24

**Published:** 2025-03-05

**Authors:** Randolph P. Matthews, Munjal Patel, Wen Liu, Yang Liu, Juan C. Rondón, Ryan C. Vargo, S. Aubrey Stoch, Marian Iwamoto

**Affiliations:** 1 Merck & Co., Inc. 2793 , Rahway, New Jersey, USA; 2 Clinical Pharmacology of Miami, Hialeah, Florida, USA; IrsiCaixa Institut de Recerca de la Sida, Barcelona, Spain

**Keywords:** islatravir, pharmacokinetics, human immunodeficiency virus, hepatic impairment, liver disease

## Abstract

Islatravir (ISL) is a nucleoside reverse transcriptase translocation inhibitor in development for the treatment of HIV-1 infection. People living with HIV are at risk of liver disease. ISL is metabolized by adenosine deaminase (ADA), which is expressed in the liver; thus, ISL pharmacokinetics (PK) may be affected by hepatic impairment. This study evaluated the effect of moderate hepatic impairment on ISL PK. This nonrandomized, open-label, phase 1 study (MK-8591-030) evaluated the effects of a single oral dose of ISL 60 mg in HIV-seronegative adults with moderate hepatic insufficiency (*n* = 6) and matched healthy adult participants (*n* = 6). Blood samples for plasma ISL and 4′-ethynyl-2-fluoro-2′deoxyinosine (M4) and peripheral blood mononuclear cell (PBMC) ISL-triphosphate (ISL-TP) were collected at multiple time points through 672 h, and safety was monitored throughout. Modest decreases in maximum measured concentration (*C*_max_) and area under the concentration-time curve (AUC) of plasma ISL and AUC of PBMC ISL-TP were observed in participants with moderate hepatic impairment versus matched healthy participants, while ISL-TP *C*_max_ was relatively unchanged. In contrast, plasma M4 was modestly increased in the moderate hepatic impairment group, suggesting that hepatic impairment may result in increased metabolism of ISL to M4 via ADA. The clinical relevance of the overall modest changes in M4, ISL, and ISL-TP levels with moderate hepatic impairment will be contextualized once exposure response data from ongoing clinical studies are available to elucidate the thresholds for clinical efficiency. A single oral dose of ISL 60 mg was generally well tolerated in both groups.

**CLINICAL TRIALS:**

This study is registered with Clinicaltrials.gov as NCT04515641.

## INTRODUCTION

HIV-1 remains a formidable health challenge worldwide, with an estimated 40 million people living with HIV and approximately 1.3 million new HIV-1 infections occurring annually as of 2023 (1). The advent of, and advances in, antiretroviral therapy (ART) for HIV over the past ~40 years have significantly improved the treatment of HIV infection (2–4). However, people living with HIV who are on long-term ART are at risk of developing chronic conditions typically associated with aging and corresponding metabolic changes, including kidney, liver, and cardiovascular diseases (2, 5). In addition to these chronic disorders, individuals living with HIV are at higher risk for liver disease from coinfection with hepatitis B and C viruses, drug-induced liver injury due to long-term ART use, and effects of other drugs and substances on the liver (6, 7). In general, the hepatic impairment associated with these conditions may affect the pharmacokinetic (PK) profile of small molecules, particularly those that undergo significant metabolism in the liver. Despite this, hepatic impairment has been shown to have no significant effect on the PK profile of several antiretroviral agents currently in use (8, 9).

Islatravir (ISL) is a nucleoside reverse transcriptase translocation inhibitor that suppresses HIV-1 replication via multiple mechanisms of action, including reverse transcriptase translocation inhibition and delayed chain termination (10–13). ISL is differentiated from other HIV-1 antiretroviral agents by its antiviral activity (notably against typically treatment-resistant HIV-1 strains), high potency, long half-life, favorable drug resistance profile, and broad pharmacologic distribution (14, 15), making it ideal for low-dose regimens. Furthermore, the PK of ISL has been demonstrated to be dose-proportional over a wide range of doses (0.25 to 400 mg) (16). Similar to other nucleoside reverse transcriptase inhibitors, ISL is rapidly converted to its active triphosphate form, ISL-triphosphate (ISL-TP), within target cells (17). ISL is metabolized to 4´-ethynyl-2-fluoro-2´deoxyinosine (M4) by adenosine deaminase (ADA), which is found in multiple tissue types, including the gastrointestinal tract, testes, lymphoid tissue, and the liver (18, 19). M4 is subsequently eliminated via renal excretion (20). ISL is not metabolized *in vitro* by hepatic enzymes such as members of the cytochrome P450 family (20), and thus a more “typical” effect of hepatic impairment on ISL and ISL-TP was not anticipated. However, possible decreases in ADA activity in the liver of a hepatically impaired individual could lead to lower M4 levels and a corresponding increase in ISL. Alternatively, increases in ADA activity due to inflammatory or metabolic liver disease could lead to higher M4 levels and a decrease in ISL (21–23).

ISL dose- and time-related decreases in total lymphocyte and lymphocyte subset counts (CD4+ T cell, CD8+ T cell, and B cell) were observed in some participants in several discontinued phase 2 and 3 trials that investigated ISL administered at higher doses (24), including 0.75 mg daily (25, 26), 20 mg weekly (27, 28), and 60 or 120 mg monthly (29, 30). Currently, lower doses of ISL are being studied for HIV-1 treatment as daily administration (0.25 mg) with doravirine (31–34) and as weekly administration (2 mg) with lenacapavir (35). Although there is no longer a monthly ISL program, the 60 mg dose was selected for this study because, at the time this study was initiated, 60 mg was the dose selected for assessment as monthly administration for pre-exposure prophylaxis. Because of the wide range of ISL dose proportionality, PK results from a 60 mg dose are likely scalable to the 0.25 mg and 2 mg doses.

There is a need to better understand whether hepatic impairment affects the PK and tolerability of ART in people living with HIV; therefore, evaluating the effects of hepatic impairment on the PK of ISL is important. Herein, the results of a phase 1 study assessing the effect of moderate hepatic impairment on the PK of ISL, ISL-TP, and the major ISL metabolite M4 after a single oral dose of ISL 60 mg are reported.

## RESULTS

### Participants

A total of 12 participants (six with moderate hepatic impairment and six healthy matched control participants) were enrolled (Table 1). The mean ages were 60.5 (range 45 to 69) and 59.5 (range 52 to 67) years for participants with moderate hepatic impairment and healthy control participants, respectively. The majority of participants with moderate hepatic impairment were White, female, and Hispanic or Latinx (6/6, 4/6, and 4/6, respectively). A similar distribution was observed among the healthy control participants (5/6 White, 3/5 female, and 5/6 Hispanic or Latinx).

**TABLE 1:** Participant demographics and baseline characteristics^*a*^

	Moderate hepatic impairment (*n* = 6)	Healthy matched participants (*n* = 6)
Sex, *n*		
Male	2	3
Female	4	3
Age, mean (SD), years	60.5 (9.4)	59.5 (6.6)
Range	45 to 69	52 to 67
Race, *n*		
Black or African American	0	1
White	6	5
Ethnicity, *n*		
Hispanic or Latinx	4	5
Not Hispanic or Latinx	2	1
Weight, mean (SD), kg	77.5 (10.1)	73.8 (5.5)
BMI, mean (SD), kg/m^2^	29.7 (3.0)	27.6 (1.4)
Range	25.6 to 33.0	25.3 to 33.0
Total Child-Pugh score^*b*^ (range)	7 to 8	NA

^*a*^ BMI, body mass index; NA, not applicable; SD, standard deviation.

^*b*^ Total Child-Pugh score was used to categorize hepatic impairment.

### Plasma ISL and M4 PK

**Fig 1:**
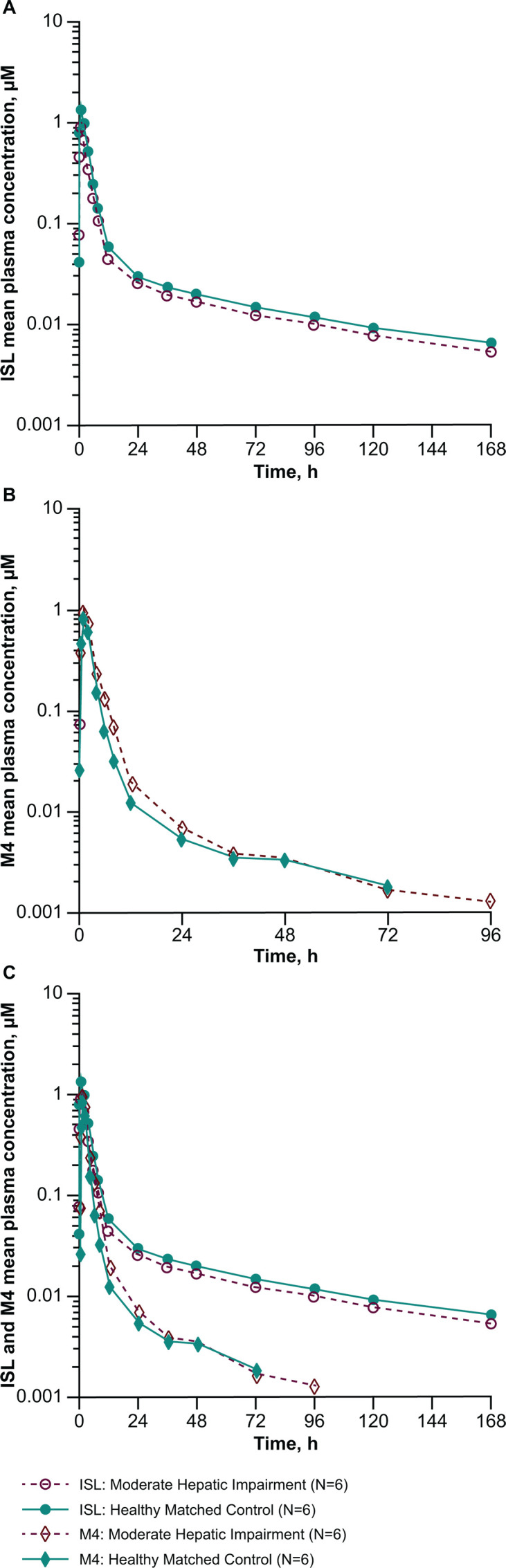
Plasma concentration versus time profiles of ISL and M4. Semi-log scale arithmetic mean plasma concentration versus time profiles of (**A**) ISL only, (**B**) M4 only, and (**C**) ISL and M4 after administration of a single oral dose of ISL 60 mg to participants with moderate hepatic impairment and matched healthy control participants (*n* = 6 per group).

The mean ISL and M4 plasma concentration-time profiles are shown in Fig. 1, and PK parameter values are shown in Table 2. After administration of a single oral dose of ISL 60 mg, participants with moderate hepatic impairment had 25%, 25%, and 35% lower geometric mean plasma ISL area under the concentration-time curve from administration to infinity (AUC_0-∞_), area under the concentration-time curve from administration to last sampling time after dose (AUC_0-last_), and maximum measured concentration (*C*_max_), respectively, relative to healthy matched control participants, although the confidence interval (CI) of the geometric mean ratio (GMR) for *C*_max_ included unity. In contrast, participants with moderate hepatic impairment had 20%, 22%, and 10% higher geometric mean plasma M4 AUC_0-∞_, AUC_0-last_, and *C*_max_, respectively, relative to matched healthy control participants, although the CIs of the GMRs included unity for all parameters for plasma M4.

**TABLE 2:** Summary of plasma ISL and plasma M4 PK in participants with moderate hepatic impairment and healthy matched control participants after administration of a single dose of ISL 60 mg^*a*^

Analyte (matrix)	PK parameter	GM (95% CI)		GMR (90% CI)
		Moderate hepatic impairment (*n* = 6)	Healthy matched participants (*n* = 6)	Moderate hepatic impairment/healthy matched participants
ISL (plasma)	AUC_0-∞_,^*b*^ h·µM	5.81 (5.08, 6.64)	7.74 (5.67, 10.6)	0.75 (0.58, 0.96)
	AUC_0-last_,^*b*^ h·µM	5.25 (4.55, 6.07)	6.97 (5.04, 9.63)	0.75 (0.58, 0.98)
	*C*_max_,^*b*^ µM	0.834 (0.440, 1.58)	1.28 (0.822, 1.98)	0.65 (0.38, 1.14)
	*T*_max_, median (min, max), h	1.00 (1.00, 6.00)	1.00 (1.00, 2.00)	NA
	*t*_½_,^*c*^ h	72.9 (13.6)	83.9 (12.0)	NA
	CL/F,^*c*^ L/h	35.1 (12.9)	26.5 (30.3)	NA
	Vz/F,^*c*^ L	3,714 (18.4)	3,197 (38.6)	NA
M4 (plasma)	AUC_0-∞_,^*b*^ h·µM	2.98 (1.73, 5.13)	2.48 (1.80, 3.42)	1.20 (0.76, 1.90)
	AUC_0-last_,^*b*^ h·µM	2.86 (1.60, 5.10)	2.35 (1.69, 3.26)	1.22 (0.75, 1.97)
	*C*_max_,^*b*^ µM	0.895 (0.427, 1.88)	0.815 (0.580, 1.14)	1.10 (0.60, 2.00)
	*T*_max_ median (min, max), h	1.00 (1.00, 2.00)	1.00 (1.00, 2.00)	NA

^*a*^ AUC_0-last_, area under the concentration-time curve from administration to last sampling time after dose; AUC_0-∞_, area under the concentration-time curve from before dose to infinity; CL/F, apparent clearance after extravascular administration; *C*_max_, maximum measured concentration; GCV, geometric coefficient of variation; GM, geometric least-squares mean; GMR, geometric least-squares mean ratio; ISL; islatravir; M4, 4´-ethynyl-2-fluoro-2´deoxyinosine; NA, not applicable; PK, pharmacokinetics; *t*_½_, apparent terminal half-life; *T*_max_, time to maximum measured concentration; Vz/F, apparent volume of distribution during the terminal phase.

^*b*^ Back-transformed least-squares mean and 95% CI from fixed-effects model performed on natural log-transformed values.

^*c*^ Geometric mean (%GCV).

### Peripheral blood mononuclear cell ISL-TP PK

**Fig 2:**
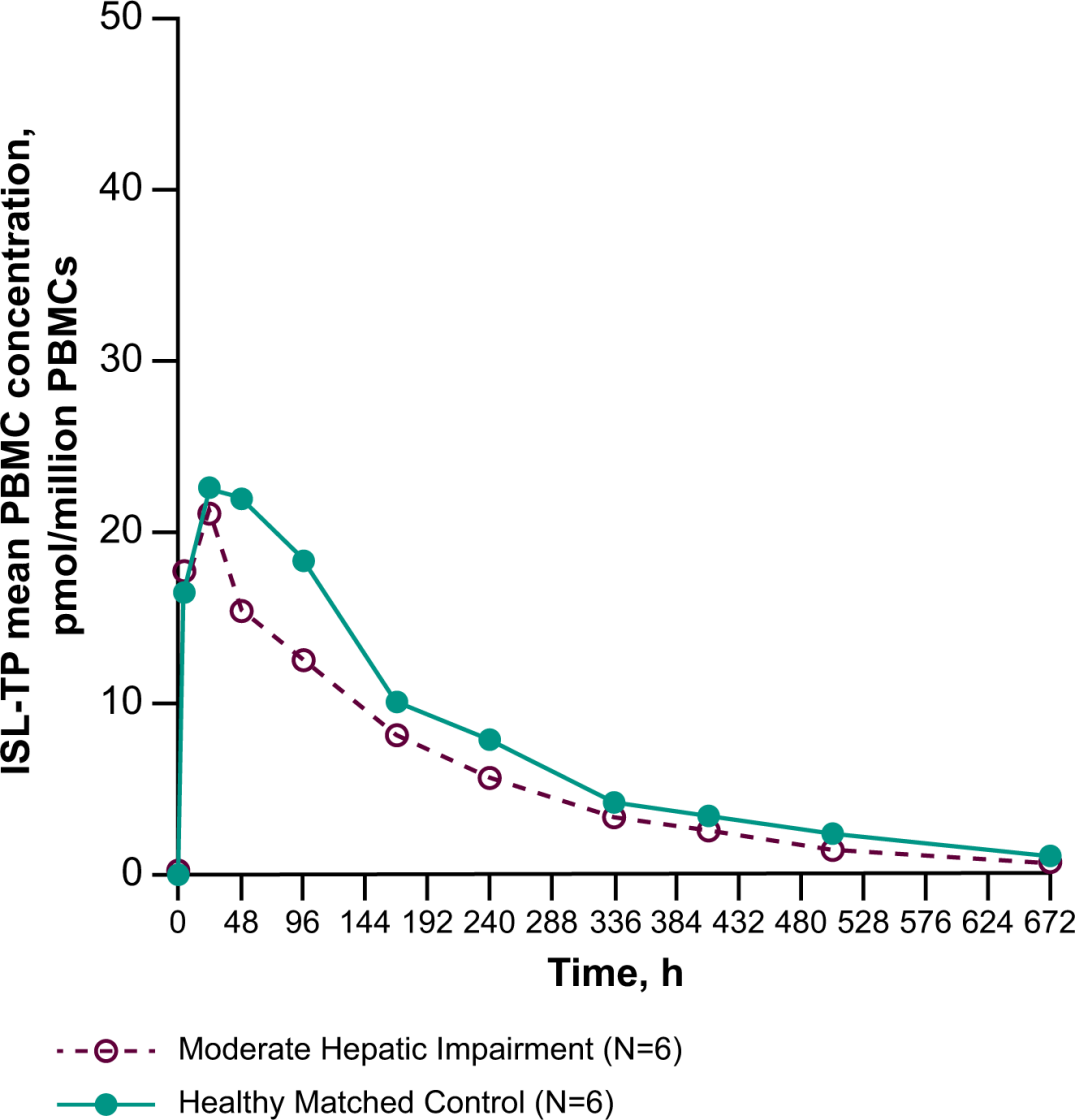
PBMC concentration versus time profiles of ISL-TP. Arithmetic mean PBMC concentration versus time profiles of ISL-TP after administration of a single oral dose of ISL 60 mg to participants with moderate hepatic impairment and matched healthy control participants (*n* = 6 per group).

The mean ISL-TP intracellular concentration-time profiles are shown in Fig. 2, and PK parameter values are shown in Table 3. Participants with moderate impairment had 24%, 23%, 14%, and 35% lower geometric mean peripheral blood mononuclear cell (PBMC) ISL-TP AUC_0-∞_, AUC_0-last_, and concentration at 168 and 672 h after dosing (C_168_ and C_672_), respectively, and similar *C*_max_ and concentration at 24 h after dosing (*C*_24_) relative to healthy matched control participants following administration of ISL 60 mg. However, the CIs for all GMRs include unity. The observed median time to maximum measured concentration (*T*_max_) and geometric mean apparent terminal half-life (*t*_½_) were shorter in participants with moderate hepatic impairment compared with the healthy matched control participants.

**TABLE 3:** Summary of PBMC ISL-TP PK in participants with moderate hepatic impairment and healthy matched control participants after administration of a single dose of ISL 60 mg^*a*^

PK parameter	GM (95% CI)		GMR (90% CI)
	Moderate hepatic impairment (*n* = 6)	Healthy matched control (*n* = 6)	Moderate hepatic impairment/healthy matched participants
AUC_0-∞_,^*b*^ h·µM	19,500 (15,200, 25,000)	25,700 (19,000, 34,700)	0.76 (0.57, 1.00)
AUC_0-last_,^*b*^ h·µM	18,600 (14,700, 23,500)	24,100 (17,300, 33,600)	0.77 (0.58, 1.03)
*C*_max_,^*b*^ µM	109 (83.7, 141)	113 (68.0, 189)	0.96 (0.63, 1.46)
*C*_24_,^*b*^ µM	103 (74.7, 141)	97.6 (54.3, 175)	1.05 (0.65, 1.71)
*C*_168_,^*b*^ µM	39.9 (30.0, 53.1)	46.4 (29.6, 72.6)	0.86 (0.59, 1.26)
*C*_672_,^*b*^ µM	3.80 (2.25, 6.42)	5.82 (4.38, 7.74)	0.65 (0.42, 1.01)
*T*_max_, median (min, max), h	24.00 (4.00, 48.00)	48.00 (24.00, 96.00)	NA
*t*_½_,^*c*^ h	148 (18.4)	169 (31.9)	NA

^*a*^ AUC_0-last_, area under the concentration-time curve from administration to last sampling time after dose; AUC_0-∞_, area under the concentration-time curve from before dose to infinity; C, concentration at hours after dosing; *C*_max_, maximum measured concentration; GCV, geometric coefficient of variation; GM, geometric least-squares mean; GMR, geometric least-squares mean ratio; ISL; islatravir; NA, not applicable; PBMC, peripheral blood mononuclear cells; PK, pharmacokinetics; *t*_½_, apparent terminal half-life; *T*_max_, time to maximum measured concentration; TP, triphosphate.

^*b*^ Back-transformed least-squares mean and 95% CI from fixed-effects model performed on natural log-transformed values.

^*c*^ Geometric mean (%GCV).

### Safety

A single oral dose of ISL 60 mg was generally well tolerated in participants with moderate hepatic impairment and in healthy matched control participants. There were no serious adverse events, discontinuations due to adverse events, or deaths reported. A total of two adverse events were reported, where one participant with moderate hepatic impairment and one healthy participant each reported headache. Both headaches were grade 1, transient in nature, and resolved within 4 h from onset. No clinically meaningful relationships were observed for changes in vital signs or clinical laboratory values, including lymphocyte counts (Table S1), as a function of the study drug.

## DISCUSSION

This study was conducted to assess the effect of moderate hepatic impairment on ISL, M4, and ISL-TP PK. The PK of plasma ISL was shown to demonstrate a modest decrease in *C*_max_ and AUC in participants with moderate hepatic impairment relative to matched healthy participants. We observed similar modest decreases in ISL-TP C_168_, *C*_672_, and AUC, while ISL-TP *C*_max_ and *C*_24_ were relatively unchanged. It should be noted that the overall exposure of both ISL and ISL-TP in the healthy participant cohort in this study was higher than that reported for healthy matched controls in a separate study assessing ISL and ISL-TP PK (36). This is most likely due to the relatively small sample size and general PK variability, resulting in distinct PK parameter values.

In contrast, plasma M4 was modestly increased in participants with moderate hepatic impairment, suggesting that hepatic impairment may result in increased metabolism to M4 via ADA. The initial, perhaps overly simplistic, expectation was that liver disease would lead to decreased ADA activity; this directional change in M4 was thus unanticipated but suggests an increase in ADA activity and/or expression, possibly due to inflammation and/or metabolic or immune dysfunction. Of note, increased ADA activity and/or expression has been observed in several liver disorders, including in patients with metabolic liver disease and inflammatory liver diseases (21–23). Thus, at least some causes of hepatic impairment can lead to an increase in ADA expression in the liver, which is further supported by the results described here. The relatively similar M4 *C*_max_ levels between hepatically impaired and matched healthy participants suggest that the effects of hepatic impairment are not related to increased gastrointestinal tract ADA expression, as an increase in gastrointestinal tract ADA would likely lead to an increase in M4 *C*_max_ in the hepatically impaired group. This further supports an increase in hepatic ADA activity and/or expression in the participants in this study. The overall changes noted for M4, ISL, and ISL-TP levels were modest. The clinical relevance of these changes will be contextualized once further exposure response data from the clinical study program for ISL are available to elucidate the significance thresholds for clinical efficacy. Preclinical evaluation of M4 suggests a low potential for host toxicity (37–40). Currently, lower doses of ISL are being studied for HIV-1 treatment as daily administration (0.25 mg) with doravirine (31–34) and as weekly administration (2 mg) with lenacapavir (35). ISL exposure after chronic daily dosing of 0.25 mg or weekly dosing of 2 mg is lower than a single 60 mg dose. Given the observed dose proportionality of ISL and ISL-TP PK, it is likely that the relative effects on these lower doses would be similar.

In this study, a single oral dose of ISL 60 mg was generally well tolerated in the six participants with moderate hepatic impairment and in the six healthy matched participants. Adverse event rates were the same in both groups (*n* = 1/6), with headache as the only adverse event reported (for each group).

Limitations of the study include a small sample size, a short exposure time, and assessment in the moderate hepatic impairment population only. The small sample size reduced statistical power, possibly impacting the interpretation of some of the PK parameters. The observations in the present study population could be interpolated to individuals with mild hepatic impairment, where a smaller effect on ISL PK would be expected. A potential effect on individuals with severe hepatic impairment cannot be extrapolated from these findings and would require additional data. Continued investigation in the ongoing clinical program to better understand the mechanisms affecting the PK and tolerability of ISL in individuals with hepatic insufficiency may further elucidate the effects expected in such populations. As mentioned, ISL continues to be investigated as part of a two-drug HIV-1 treatment regimen with doravirine (31–34) and with lenacapavir (35). Data from other clinical trials suggest that hepatic impairment does not affect the PK of doravirine (41) or lenacapavir (42).

### Conclusions

In the current study, the administration of a single oral dose of ISL 60 mg modestly decreased plasma ISL exposure and intracellular ISL-TP AUC in participants with moderate hepatic impairment, while ISL-TP *C*_max_ and *C*_24_ levels remained relatively unchanged. In contrast, modest increases were seen in plasma M4 metabolite exposure in participants with moderate hepatic impairment. A single oral dose of ISL 60 mg was found to be generally well tolerated in all participants.

## MATERIALS AND METHODS

### Study design

This was a nonrandomized, multigroup, open-label, single-dose, phase 1 study (MK-8591-030, NCT04515641) of ISL in HIV-seronegative adult participants with moderate hepatic insufficiency (*n* = 6) and matched healthy adult participants (*n* = 6).

### Participants

Male and female HIV-seronegative participants between the ages of 18 and 75 years with a body mass index of ≥18.5 and ≤40 kg/m^2^ were eligible for enrollment. Participants with hepatic insufficiency were recruited first, and healthy participants were subsequently selected so that their individual ages and weights were within ±10 years and ±10 kg of the mean age and weight of the participants with hepatic insufficiency.

For inclusion in the hepatically impaired group, participants were required to have a diagnosis of chronic (>6 months), stable hepatic disease due to any etiology, with features of cirrhosis defined as “moderate hepatic impairment” per the Child-Pugh scale (score of 7–9; Table S2). With the exception of hepatic impairment, participants were required to be in good health. Participants in the healthy matched control group were required to be in generally good health, with no chronic medical issues and normal screening electrocardiograms, vital signs, and laboratory values.

### Procedures

Participants received a single oral dose of ISL 60 mg. An ISL dose of 60 mg was assessed in this study because a 60 mg monthly dose was the highest dose being studied in phase 3 trials at the time the current study commenced. Blood samples for plasma ISL and M4 and PBMC ISL-TP were collected pre-dose and at multiple time points through 672 h after dosing for PK assessment. Safety was monitored throughout the study by repeated clinical and laboratory evaluations.

### PK analysis

ISL and M4 concentrations in plasma samples were analyzed using liquid-liquid extraction and salt-assisted liquid-liquid extraction, respectively, followed by liquid chromatography with tandem mass spectrometry. ISL-TP concentrations in PBMC samples were analyzed using protein precipitation followed by ultra-high performance liquid chromatography with tandem mass spectrometry. The lower limits of quantification were 20.0 pg/mL for plasma ISL, 100.0 pg/mL for PBMC ISL-TP, and 0.5 ng/mL for plasma M4.

The PK parameters assessed for plasma ISL were AUC_0-∞_, AUC_0-last_, *C*_max_, *T*_max_, apparent terminal *t*_½_, apparent clearance after extravascular administration, and apparent volume of distribution during the terminal phase. The PK parameters assessed for PBMC ISL-TP were AUC_0-∞_, AUC_0-last_, *C*_max_, *C*_24_, *C*_168,_ *C*_672_, *T*_max_, and *t*_½_. The PK parameters assessed for plasma M4 were AUC_0-∞_, AUC_0-last_, *C*_max_, and *T*_max_.

### Safety assessments

Safety assessments, including physical examinations, vital signs (heart rate and blood pressure), electrocardiograms, and clinical safety laboratory tests (hematology, serum chemistry, including kidney and liver function tests, and urinalysis), were monitored at the screening visit, pre-dose, and at 24 and 168 h after dosing.

### Statistical analysis

For each of the following PK parameters, individual values were natural log-transformed and assessed with a linear fixed-effects model with a fixed effect for each population (participants with moderate hepatic impairment and healthy control participants): plasma ISL AUC_0-∞_, AUC_0-last_, and *C*_max_; PBMC ISL-TP AUC_0-∞_, AUC_0-last_, *C*_max_, *C*_24_, *C*_168,_ and *C*_672_; and plasma M4 AUC_0-∞_, AUC_0-last_, and *C*_max_.

To allow for unequal population variance, an unstructured covariance matrix was utilized. The 95% CIs for the least-squares means were constructed for each population on the natural log scale and referenced to the *t*-distribution. To obtain geometric least-squares means with corresponding 95% CIs on the original scale, the least-squares means were exponentiated. A two-sided 90% CI for the true difference in means between the moderate hepatic impairment group and healthy control group was calculated for each PK endpoint, using the mean square error from the model and referenced to the *t*-distribution. To obtain the 90% CIs for the true GMR between the populations for each PK endpoint, the confidence limits were exponentiated.

## Data Availability

The data sharing policy, including restrictions, of Merck Sharp & Dohme LLC, a subsidiary of Merck & Co., Inc., Rahway, NJ, USA (MSD), is available at https://trialstransparency.merckclinicaltrials.com/policies-perspectives.aspx. Requests for access to the clinical study data can be submitted via email to dataaccess@msd.com.

## SUPPLEMENTAL MATERIAL

Supplemental tables aac.01553-24-s0001.docx Tables S1 and S2.

ASM does not own the copyrights to Supplemental Material that may be linked to, or accessed through, an article. The authors have granted ASM a non-exclusive, world-wide license to publish the Supplemental Material files. Please contact the corresponding author directly for reuse.
