# Supplementary material for: Pharmacokinetics of islatravir in participants with moderate hepatic impairment
Source: Antimicrob Agents Chemother. 2025 Mar 5;69(4):e01553-24. doi: 10.1128/aac.01553-24 (PMC11963540; doi:10.1128/aac.01553-24)
Supplement: Supplemental tables — Tables S1 and S2. [file aac.01553-24-s0001.docx]

**SUPPLEMENTAL MATERIALS**

**TABLE S1**. Summary of observed changes in total lymphocyte counts in all participants as treated

| **Time** | **Value GM (SE), 10^9^/L** | | **Percent change from baseline GM (SE)** | |
| --- | --- | --- | --- | --- |
|  | **Moderate hepatic  impairment**  **(*n* = 6)** | **Healthy matched  control**  **(*n* = 6)** | **Moderate hepatic  impairment**  **(*n* = 6)** | **Healthy matched  control**  **(*n* = 6)** |
| Predose | 1.03 (0.22) | 1.74 (0.20) | NA | NA |
| 24 h post-dose | 0.93 (0.19) | 1.55 (0.23) | –9.83 (12.77) | –10.87 (7.23) |
| 168 h post-dose | 1.07 (0.23) | 1.76 (0.20) | 3.36 (8.38) | 1.07 (6.22) |

GM, geometric mean; NA, not applicable; SE, standard error.

**TABLE S2**. Child-Pugh scale to classify severity of liver disease^a^

|  | **Points score for increasing abnormality** | | |
| --- | --- | --- | --- |
| **Assessment** | **1** | **2** | **3** |
| Encephalopathy^b^ | None | Stage 1-2 | Stage 3-4 |
| Ascites | Absent | Slight | Moderate |
| Albumin (g/dL) | >3.5 | 2.8-3.5 | <2.8 |
| INR | <1.7 | 1.7-2.3 | >2.3 |
| Bilirubin (participants without PBC; mg/dL)^c^ | <2 | 2-3 | >3 |
| Bilirubin (participants with PBC; mg/dL)^c^ | <4 | 4-10 | >10 |

INR, international normalized ratio; PBC, primary biliary cirrhosis.

^a^Points from each row are summed to give a final Child-Pugh score, where scores of 5-6, 7-9, and 10-15 are classified as mild, moderate, or severe hepatic insufficiency, respectively.

^b^Portal-systemic encephalopathy staged 0-4.

^c^Use only one based on type of cirrhosis.
